# Proxy Users Enable Older People Creative Writing on the Web

**DOI:** 10.3389/fsoc.2019.00015

**Published:** 2019-04-16

**Authors:** Piotr Toczyski, Jarosław Kowalski, Cezary Biele

**Affiliations:** ^1^Faculty of Applied Social Sciences, Institute of Philosophy and Sociology, Maria Grzegorzewska University, Warsaw, Poland; ^2^Laboratory of Interactive Technologies, National Information Processing Institute, Warsaw, Poland

**Keywords:** technology and older people, gray digital divide, creative writing, social inclusion, artificial intelligence, digital wisdom, voice assistants, participatory action research

## Abstract

This paper presents several state-of-the-art concepts within Internet studies and applies them to the creative writing of older people using the Internet. For more than 10 years two creative Web users aged 80+, assisted by younger proxy users, were involved in preliminary action research. It was aimed at finding patterns of inducing older people's creativity and sharing their wisdom with the general Internet audience. The effectiveness of conducted action research in transferring wisdom using silver digital content is high. It is demonstrated with (a) qualitative participants' insights, (b) the quantitative description of statistics of blog visits, and (c) the social significance of the topics covered in the created content. Lasting for more than a decade and located within the space of socio-technological solutions in Central and Eastern Europe, the results delivered patterns of emerging technologies aimed at enhancing older people's creativity on the Web. The insights from those two action-based case studies enabled the development of new hypotheses. New directions of further, more advanced research of older users' activity are based on interdisciplinary studies at the crossroads of public health, sociological theory, gerontology, and human-computer interaction studies. New research questions are presented, to be explored within the social scientific studies of the next-generation Internet. Departing from the established concepts and preliminary research, the authors hypothesize that: (1) in order to optimize non-human technology-based assistants, human proxy users should be researched; (2) voice assistant technology could become the primary proxy for a production of silver digital content; and (3) interactive and intelligent technology will be the substitute for social actors that prevent exclusion and disengagement. The remaining research question also refers to the conditions under which the technology can be a viable substitute for proxy users.

## Introduction

The aging population is not sufficiently self-represented in social media. Why do senior citizens not write and publish online, and in effect disengage from larger digital public sphere? In terms of disengagement and activity, there should be space for consistent and enjoyable activity in the lives of aging people.

This article broadens the discussion on the means by which to prevent digital disengagement, already started in a semi-peripheral Eastern European context. The results of preliminary action research on the creative writing and publishing of older people are helpful in developing the concept of so-called “silver digital content,” first presented in literary studies focused on creative writing (Toczyski, 2017), and transferring it to the area of social sciences. There is no lack of proven opinion leaders among senior citizens, but there is a lack of digital skills and in effect unavailability of social media interfaces for older people. As long as technological innovation and entrepreneurship cannot sufficiently respond to this challenge, social innovation can fill the gap. The role of inclusive design in making social innovation happen has already been noticed. The concept of a digitally wise human, capable of using others' digital skills to achieve their goals online has been developed within Internet studies. Departing from innovation and digital inclusion studies, the idea of including successful and productive seniors to the social Web is developed.

Writing further on silver digital content (rather than gray content) and discussing the procedure for its development and publication, we reveal our attitude to the research problem posed, in accordance with the approach adopted in research in action. Namely, we think that older people can be great creators of textual (and perhaps logo-visual or audiovisual) digital content. By metaphorically coining this term with reference to the color of silver rather than gray, we try to appreciate the postulated digital presence and digital wisdom of people in the last stages of life: advanced maturity and old age.

Creating such metaphorically described content can be achieved with the cooperation of helpful proxy users and older people. It enables older users to develop their creative writing on the Web. Theorizing such cooperative exchange in terms of digital wisdom leads to the action research report, which describes the research that has been conducted for over 10 years.

A large part of the article will be devoted to the description of how older people currently publish content on the Internet. This is done through another person, a proxy user. The digitally wise human does not have to be a digital native as even a digital immigrant can achieve digital wisdom. For people aged 85 or more and publishing on the Web (digital sages 80+) creative writing results in social media connection and new media inclusion. However, it is mainly an expression of intergenerational transfer in which seniors' wisdom matches the digital competences of their younger cooperators. Such a model of seniors' creative writing on the Web is illustrated by action research and metaphorically named silver digital content.

Proxy users have heard from participants that this digital work has an essential function in their lives, including a therapeutic and compensatory function. They receive motivating feedback from readers, often further inspiring, challenging and stimulating them to maintain their intellectual readiness.

Why do we write about proxy users, when there are already technological devices such as smart assistants? Because their functionality is limited as there is no software yet to create a voice and have a blog written and published. We want to show why this is the direction that the development of technology should take.

We are talking about a barrier that we want to overcome in practice, not just in theory. The article will, therefore, help those responsible for the design of new technologies and human-computer interactions.

We want to show what is still missing: what functions the imagined assistant lacks, so that older people can simply speak to the assistant and after 10 min have a Facebook entry and a discussion with the public. Our thoughts are focused on the fact that assistants could in the near future take over the role of technological facilitators for older people, for example, proxy users.

Business circles are already working on developing similar technologies, and soon voice interfaces will be ready and will even improve the creative use of Facebook or other social media spaces. This article indicates new dilemmas and directions of development of technologies for older people.

After describing the issues of the gray digital divide and digital wisdom created by tandems of older users and younger intermediary users, we present a report on a study in action, which may serve as a pilot study for people interested in improving the creative well-being of older people.

We write that voice interfaces combined with artificial intelligence (AI) pose new challenges and questions. To what extent should the AI interfere with an older person's writing style? Press interviews are not literal quotations either; the journalist prepares them. To what extent should technology interfere with the content? The assistant knows more quickly than an older artist what he or she wants to say, although the creator does not find the right words. As a grandson may suggest to his grandmother, when she cannot find the right words, technology might assist her in doing so. We will also raise the question of the extent to which the assistant can be given a topic, for example, to write it in such a style that it evokes emotion such as when AI uses words that increase the chances of a recipient being emotionally moved. Ultimately, the question is whether the roles will turn around at the end of the process. Older people will need technology as a generator of interesting threads in content and wisdom, but the message itself will be prepared by AI. AI will collect and search for wisdom among people and pass it on to others.

However, we start with basic conceptual categories and research in action that has lasted over a decade.

Let us begin with an obvious statement, but rarely expressed. One of the few who formulated it is the media expert (Brake David, 2014, p. 595, 598). He states that although older people have many more experiences which they might want to share; it is young people who are more intense creators of Internet content than older people. Developing this idea, it can be added that the multiplicity of experiences that older people might want to share is already derived from their own birth certificate. With time, experiences accumulate, and this accumulation could encourage the sharing of some of them—subjectively considered to be particularly worth telling.

Anticipating the metaphors present in the literature, which we would like to recall, one could say that older people—figuratively: gray (gray-haired)—do not take on the role of digital sages, because they feel that they are digital immigrants—people with a lower status in the digital world than digital natives. Meanwhile, in cooperation with natives, they could achieve the ideal of digital wisdom and offer silver content to online readers. Only this last slogan—silver content—is the new complement to the metaphors already well-established in the literature on Internet science. The author of the digital wisdom is Prensky (2009), who, after several years decided that to divide netizens into age groups is insufficiently accurate. He decided to complement it with the concept of digital wisdom, which can become a part of both groups: younger and older.

We place our reflections on the above topics in the analysis of several types of exclusion: media, information, and digital. In the literature one can find the term media exclusion. What does it mean? The authors usually understand this phenomenon as excluding a group from the media content or selection of topics presented in the media, when one group gets much more attention than others (Zbyrad, 2013, p. 98). We suggest enriching these two terms with a statement that media and information exclusion can take the form of not only the consumption of media content, but also their production. At a later point in this paper, we will focus on the issue of exclusion from the production of the content, of new media, and on creative writing and cooperation in publishing as well as its effect as a mechanism to counteract exclusion.

## Between the Gray of the Digital Divide and the Silver of the Digital Economy

The British scholar Selwyn (2010) uses the concept of the digital divide. However, in earlier works, he (Selwyn, 2004) was one of the few who asked the question about the validity of the use of the term “divide” or “exclusion” in relation to Internet technology. He noted that as early as the nineties political discussion in the Western world focused on the haves and have-nots of information, that is, people with or without information resources. At that time, the concept of informational and communicative poverty also appeared, but over time the most popular slogan became the concept of the digital divide, or digital separation (Selwyn, 2004, p. 344).

What are the sources that divide those into the excluded and those actively using information technology? Social resources are conducive to the active use of technology. As Selwyn says, we should note that when an individual has obtained the right conditions for access to different technologies, the lack of significant use of them is not necessarily due to technological factors (such as lack of physical access, skills or operational efficiency) or even psychological factors (such as restraint or anxiety related to the use of technology), as it is generally accepted by technologists (Selwyn, 2004, p. 349). So, what affects the involvement of individuals in the use of information and communication technologies? Selwyn responds with a complex mix of social, psychological, economic, and above all pragmatic reasons (Selwyn, 2004, p. 349)—to what extent people can use social resources to make access useful (Selwyn, 2004, p. 349). Physical access to the technology itself—which we often forget—is not yet a social resource.

Now, let us discuss old age. Old age is conceptualized as a gray period of life, and commonly metaphorically referred to as the “gray tsunami”. If the great wave is to flood a land, then this land—it seems—is less numerous in Western societies, than young people. The younger ones are to be flooded by a wave of seniors (why, however, destructive?). In 2003, Peter Millward, then a British student and volunteer working for the social organization Age Concern, used the term gray, digital divide. In this way, he referred to the social digital divide he observed, which resulted in the declared disinterest of older people in using the Internet.

This exclusion of older people from the Internet space was associated with the lack of sufficient skills to use the Internet. This is at least partially due to a fact that a typical WIMP (windows, icons, mouse, and pointer) paradigm seems to be problematic to use by older adults because of, for example, their lowered motor control, which may cause problems in operating a mouse as well as the need to get acquainted with the vast set of concepts connected with current standard computer or mobile devices (such as folders, files, or programs). The step in the evolution of computer systems that encouraged older adults to use computers and especially touchscreen devices was the development of speech-to-text technology, and in effect, voice search and voice typing, which has been rated positively by users (Kumar et al., 2012). In effect, these technologies have become very popular in recent years, and not only among older adults. Currently, disconnecting voice search from a smartphone, and enhancing it with AI creates a product (namely voice assistant) potentially very interesting for older adults, which is connected to Brandtzaeg and Følstad (2017) conclusions that unlike other devices and software, conversational agents ought to be created as tools, toys, and friends at the same time. Such devices can become facilitators of silver digital content creations in the near future. Because they use a natural voice as their interface, voice-assistant devices are also very interesting from this perspective, because they are one of the few examples where technology itself can be a means to lowering the digital divide.

On the other hand, the declaration of lack of interest in participating in online activities, is an expression of reluctance to admit to others that such skills are lacking. Peter Millward was the first to publish his observations under the slogan “gray digital divide” (Millward, 2003). Moreover, even though in his later career he did not study this subject scientifically, his work was noticed, and the metaphor of the gray digital divide began to be used by other authors as well.

It seems that this metaphor carries a certain amount of kindness for older people, but it also implies a problem. What is gray—it is problematic, because it is either non-descript (mediocre) or not fully unambiguous (such as “gray zone”). Meanwhile, the metaphor of the silver economy has already settled in two cycles: academic and public policy analyses, referring to silver as a symbol of old age when talking about the purchasing power of seniors. Silver is to be an economy in which consumers—but less often producers—are older people. What is silver? According to Klimczuk (2011), it refers primarily to senior-oriented and gerontechnology-oriented, understood as a new scientific-research and implementation paradigm.

One could consider for a long time whether the deep source of this metaphor is merely an image associated with age reflected by gray hair, or something more. When talking about the silver economy, the question about the silver digital economy should appear immediately when considering Internet science. This is a question about older producers, brokers, and consumers in the online environment. Old age in combination with the Internet is sometimes imagined in the form of silver surfers Choudrie et al. (2013). Before that, the concept of silver surfers has already appeared in other work by Jyoti Choudrie, written with Susan Gray and Nicholas Tsitsianis—on how to encourage older people to use the Internet (Choudrie et al., 2010). On the other hand, it is almost impossible to find links between the old and the silver in old age, with reflection on the work of older people and the digital content they produce. As the Internet can be used to stay active and engaged in the social interactions, for example, through social media, we could assume, according to the activity theory of aging, that silver content creators' quality of life will be enhanced thanks to participation in the new media.

## Wisdom and Digital Wisdom

Let us now consider more closely the most-known metaphor of the digital divide: the distinction between digital natives and immigrants in comparison with digital wisdom. The author of this very popular distinction of people as native digital and inflow to the digital world is Prensky (2001a,b). The distinction, which appears in over ten thousand texts, has long been associated with its author. Separated from him, it remains one of the most widely known snapshots between Internet users of all ages. However, following the history of this concept in Prensky's work, we reach the third and least known concept from his theory—the category of digital wisdom.

For the sake of sufficient deliberate consideration of digital wisdom, we first need to know what wisdom is as such. So, before we discuss digital wisdom, let us look at wisdom.

It turns out that the unambiguous and commonly shared definition of wisdom is simply lacking, and even the link between wisdom and old age is not apparent and unambiguous in the literature. Nevertheless, although the concept of wisdom has not been agreed on so far, it is used colloquially and academically, with the intention of emphasizing the positive, beneficial aspects of acquiring life experience.

As Susanne König and Judith Glück wrote in an encyclopedic entry on the topic of academic wisdom, researchers are still arguing about its universal definition. They are only in agreement on two points. First of all, wisdom is to be an ideal final state of human development, which is fully achieved by human beings with difficulty—if at all (König and Glück, 2014, p. 7143). Second, researchers agree that wisdom is multifaceted, as it concerns the cognitive, emotional, and motivational sphere. On the other hand, the distinction and the disagreement over it concerns, above all, general wisdom—the knowledge and understanding of life in general (König and Glück, 2014, p. 7143)—and personal wisdom, or knowledge and understanding based on personal experience throughout the course of life (König and Glück, 2014, p. 7143).

Regardless of this distinction, it can also be assumed that there are two paths to defining wisdom. The first is the cataloging of the components of wisdom. Glück and Baltes (2006) recall the decades of research by the team of Paul B. Baltes on the psychology of wisdom, resulting in the so-called Berlin model of wisdom or the Berlin paradigm of wisdom. In this model, wisdom is defined as expert knowledge about the basic pragmatics of human life. The criteria for measuring knowledge related to wisdom were developed in the course of research, and the result of this reflection is an attempt to identify interventions that encourage individual expression of knowledge associated with wisdom (Glück and Baltes, 2006, p. 686). These interventions are simple and boil down to asking respondents to try to provide a wise answer to the dilemmas presented to them (Glück and Baltes, 2006, p. 686). In the second approach to defining wisdom—referred to by König and Glück (2014)—the authors understand it as referring to the integration of different dimensions of personality: those connected with the cognitive, reflective and affective function of the mind.

Interestingly, wisdom is not necessarily bound to old age—and there are even arguments for the opposite. Edmondson (2013, 2015), an Irish researcher of issues related to old age and wisdom, writes in the syllabus of her university seminar on aging, life course and sociology of wisdom, that for millennia, human aging has been associated with the development of wisdom— based on accumulated opportunities, hidden, or open, responding to other people, to oneself and deep problems of human existence, for which there is no answers (Edmondson, 2013). Achieving this socially recognized state is supposed to balance the emotional pain of aging, but also to increase the possibility of older people participating in social life. Therefore, wisdom appears as a factor that alleviates old age and as a valuable resource offered to the public by older people. Furthermore, Edmondson says that older people are expected to be wise, but the concept of wisdom becomes unclear, which makes it more difficult for older people to respect themselves (Edmondson, 2013). In this brief observation of Irish social reality, there is a statement of change in the social perception of wisdom and old age. Both the image of older people ceases to be clearly associated with wisdom, and the image of wisdom ceases to have evident attributes—to the extent that it is not even always known to what it refers to.

Traces of this approach can already be seen in the previous literature. Meacham (1990) recognizes that wisdom does not have to be associated with old age and presents a long discussion on this subject. It can be summarized as follows: older people do not like to speak about their competences in terms of wisdom; they are more distanced from determining their life experience as wisdom. At a younger age, people tend to describe older people as wise. Nevertheless, older people are also forced to use the category of wisdom in their thinking. Why? In a society where entering old age has for centuries meant entering the risk of losing control over resources in your life as well as losing power, respect, and status, it is reasonable to believe that some special properties have been achieved that in the course of life younger people cannot break away from (Meacham, 1990, p. 197). Among these properties are learning through experience, being mature and respected, being a source of good advice—but also knowing when to stop yourself—and understanding others and being interested in them. The list also includes features such as philosophy, insight, and discernment. Such a social perception of wisdom also includes the appreciation and valorization related to old age. These are features that are entirely unattainable other than through six or seven decades of professional and emotional life.

However, Meacham complicates the picture, indicating that this experience is the greatest threat to wisdom, especially if it only leads to the accumulation of information, success and power (Meacham, 1990, p. 209). Thus, experience can also lead to the risk of losing wisdom, which may occur, for example, in the social atmosphere of the growth of stereotypes and intolerances or during the sudden technological or cultural change. Therefore, with increasing age, —one must take more effort to preserve wisdom. This is a unique view, showing the complexity of linking wisdom with age. It is particularly worth remembering the idea that it is difficult to attribute wisdom to all older people, and the development of technology can lead to seniors losing of wisdom.

As Meacham says, only a rather limited and unique set of experiences can be helpful in maintaining or restoring wisdom, and these experiences take place in an atmosphere of safety, as part of a supportive interpersonal relationship where people can safely discover and reveal limitations and doubts, concerning what they know (Meacham, 1990, p. 209). Such an atmosphere is conducive to shaping wisdom. It is not the essence of wisdom—Meacham says—that the quality of our words and deeds change to more complex, sophisticated, or even deep words and deeds, because experience, and maturity only changes the quality of how wisdom is expressed—from simple to profound words—without touching the heart of wisdom (Meacham, 1990, p. 209). The core of this is to have an attitude that combines the belief that knowledge is unreliable with the struggle for a balance between knowing oneself and doubting oneself. One would think that this is a call to cognitive prudence—but is undoubtedly beyond measure. Excessive doubts is a factor unfavorable to wisdom.

When we look at the functioning of the notion of wisdom in literature, let us assume that wisdom is where people from a given population are relatively unanimous in recognizing it. A person that is considered wise will be such in perception and opinion of individual people or social groups. The measurement of wisdom is difficult, for example, because measuring tools would have to be made by people who are wiser than those subject to measurement. However imperfect this conclusion is, it seems sufficiently concise with the further goal—to understand digital wisdom.

## The Digital Wisdom of an Immigrant

Let us return to the question of whether you can be digitally incompetent but digitally wise. In 2009, when the debate on binary digital divides had not yet been heard, Marc Prensky faced such a question. Prensky was already known at the time for introducing concepts that were well-suited to academic and social needs. In 2001, he used the distinction between digital natives and digital immigrants, which then spread widely in the debate on the development of the Internet in academic circles and among practitioners (Prensky, 2001a,b). That short work in two episodes—as the citation index indicates—was referenced in over ten thousand other texts over the past decade. These were often critical texts about the simplified distinction used by Prensky (Helsper and Eynon, 2010), and this opposition itself is sometimes described as dangerous (Bayne and Ross, 2007).

It cannot be doubted, however, that this distinction is one of the best known, where the Internet is mentioned with reference to the dissonance between different age groups. The author himself later developed his concept, writing about, for example, the immigrant accent of newcomers to the Internet (Prensky, 2003). After 10 years, in the collective volume on the deconstruction of the digital natives, Prensky has already clearly associated his divide with digital wisdom (Prensky, 2011). This concept was used for the first time 2 years prior—in a work titled with various metaphors; digital intelligent human, digital natives, digital immigrant, and digital wisdom (Prensky, 2009).

Undoubtedly, through these metaphors, Marc Prensky opened up new horizons for meditations on the Internet—if he did not say anything new, at least he said it more understandably, and thus submitted to a wider and more penetrating discussion. Let us try to look at the concept of digital wisdom and how it is understood in literature.

It is worth noting that although this is a discussed concept, it seems to arouse less interest (also critical) than the divide into online natives and newcomers. This was criticized by scholars as almost offensive (Brown and Czerniewicz, 2010). There are geographical spaces in which the word native does not refer to superiority or the future, but rather to what is lagging behind and civilized only by settlers. Critics note that Prensky first used these colonial metaphors, which he later replaced with an evolutionary metaphor: homo sapiens digital. This, in turn, implies superiority, progress, advantage, and prospects for those who evolve in the digital direction—but also stagnation for those who do not evolve. It is an assumption that people are born into something that determines them and cannot change them—it is also problematic for critics (Brown and Czerniewicz, 2010). On the other hand, there are also authors who read Prensky's idea differently: Originally pushing away older people, and especially older teachers, as digital immigrants who have to try to adapt to the use of digital technologies, Prensky admitted finally that they could aspire to achieve “digital wisdom" (Richardson and Jelfs, 2015, p. 91).

So, emerges the third concept next to the native and immigrant, which should be labeled as a digital sage. Although Prensky (2009) used the slogan homo sapiens digital, digitally wise human, or digital sage seems more accurate. It goes here not only for thinking and the resulting efficiency in the use of digital technologies but also for the general capital of wisdom, only taking into account this digital efficiency. Therefore, it is digital wisdom, not digital thinking which is helpful in understanding the relationship between the two processes of immersion—deepening—in the Internet space: between digital media education of natives and the digital inclusion of immigrants. The slogan “digital wisdom” supplements the processes of digital education and digital inclusion with the intergenerational transfer of a potentially valuable resource: wisdom.

The path of digital immigrants to digital proficiency deserves special attention. The internship of digital natives in the use of the Internet is often longer even though they are younger people: in the situation of the rapid development of technology they gain experience in the course of their lives with the Internet—and thus experience—at a much earlier stage than older people. Older people—who in turn, as Biller (2013) states, create a gray digital divide—represent an inflow to the digital world of the population or immigrants.

Digital immigration is still ongoing. Tim Riley notes that in Britain the increase in the use of digital technologies—including the Internet—among pensioners is on the rise. The majority use the Internet according to the consumption pattern—they search for and consume online goods and materials (Riley, 2013, p. 51). Some, however, exhibit a creative pattern, providing their own contribution. They create and share their own content. Riley looked at the online activity of people over the age of 65 and said that some of them are learning digital technology even without intending to get closer to technology. This is a consequence of changes in interests during retirement, such as a new hobby or a re-deepening of the hobby once abandoned due to lack of time. Riley calls this phenomenon re-education of retired content creators in the field of digital technology, following their own initiative.

One would think that although an older person delivering their own content is a digital immigrant, in certain circumstances they can achieve digital proficiency. This is an observation coinciding with the proposal of three authors—Wang et al. (2012)—who propose to move away from the divide into digital natives and digital immigrants. Instead, they propose to adopt the concept of digital proficiency, which would be the ability to reform knowledge and produce information in order to express themselves creatively and appropriately in the digital environment (Wang et al., 2012, p. 409). However, can digital proficiency lead to an increase in the amount of Internet content created and shared by older people on the Internet? Schradie (2011) notes the class gap between producers of Internet content, and the critical mechanism of this inequality is the control of digital tools and the elite habitus, the element of which is the use of the Internet—and information in general (Schradie, 2011, p. 165). The creators of online content differ in terms of resources and opportunities they have—and the class is a differentiating factor. This is the thesis about the equality nature of the Internet. However, the social class is not related to age; different age categories are represented in various social classes. In other words, a higher social class can promote digital proficiency even in older age. Hence, a digitally proficient native does not have to be digitally wise, and a digitally wise immigrant does not have to be digitally proficient. This lack of digital competence and the lack of wisdom can, however, complement each other, leading to the creation of an optimal situation.

The figure of the wise human and his or her wisdom requires determining the place of the latter in the structure of life goals or side effects of the unplanned effects of life. Let us remind that wisdom is often understood as what we are aiming at. Considering the concept of wisdom as such—not digital—an observation was made that very few people are seen as wise. Paradoxically, a wise human is one who knows that the ideal of wisdom is in principle unattainable. This definition, however, does not apply without a valid reason, and when the community considers someone to be wise, it is assumed that wise is the one who was not only in the process of gaining knowledge but is also a resource of life experience. Thus, if we ask who is more likely to bring wisdom to the online communication space, we would point not at digital natives, but at immigrants.

By formulating the above assumption, we make a reservation here that linking wisdom with age is only probable and certainly not absolute. Although there are many people who are colloquially perceived as “old fools,” the social understanding of wisdom suggests a multitude of experiences as a condition—(a necessary, though insufficient) wisdom. Life experiences and reflections around them usually accumulate over time. The above mentioned phrase in socio-lingual circulation expresses a certain disappointment with the lack of archetypal wisdom in old age. It can, therefore, be assumed that the wise old man is an archetype, while the wise young man is an exception.

However, with the development of technology, many experiences become less useful—and older people usually cannot use new technologies freely. Still, more often than young people, they have the knowledge or even wisdom flowing from experience in non-internet spaces of social life. This situates them in both a pre- and post-figurative culture, in which they have resources unavailable to young people.

Is it possible to complicate this picture, including the features of a collective culture in which exchange takes place between peers? There are certainly old digitals who can help each other know about effective and active functioning in the new media space. There are also digitally incompetent young people. However, here we focus only on this segment of social reality—in these situations—where we deal with digitally competent young people and digitally incompetent or even excluded seniors. Even if this class of social phenomena was rare, it is worth formulating a particular program of activities for the digital inclusion and solution of even a rare social problem. The proposal of such a program is a suggestion of the solution to the above dilemma through the cooperation of the digitally indigenous and immigrants in tandem.

What would such cooperation require? Probably a bilaterally active—even proactive, with initiative—commitment, and understanding of the above diversity by both parties. A digitally wise human can creatively find himself in the space of the Internet, not so much because of their fluency in using hardware and software that publishes content, but mainly through the interesting content resulting from the experience to convey. Sharing by such experiences of immigrant-seniors is possible through the cooperation of digital natives who could handle hardware and software tools for them. Moreover, the goal of such activity is creative self-expression and maximization of the manifestations of wisdom available on the Internet. The condition for the existence of such cooperation, however, is the awareness of the importance of the digital world in the hierarchy of the digital immigrant's values—even though their decision is to engage in Internet communication, and not, for example, writing letters to the editor.

## Methods

Although this hypothesis paper is mostly conceptual, aimed at developing hypotheses and concepts in terms of interdisciplinary theoretical frameworks, the authors wish not to remain groundless. In order to do so, we will illustrate this concept with several points from the action research report, with reference to research that one of us has conducted for 10 years. Our goal in this part of the text is to recall the research procedure in order to illustrate the problem that has been discussed above in theoretical terms. We use the phrase “silver content,” and adopt the study in action method for this reconnaissance. We do not present the full report here but only general comments on the course of the research and main findings. We want to show that silver content requires a lasting and satisfactory existence of at least three elements: design decisions, stimulation, and active support in essential supporting activities.

The term silver digital content has probably been used only once before (Toczyski, 2017). This conclusion has been drawn from the literature review, as presented in the first part of the text. This new term is based on the proxy user's reflections on the participation of older people in the digital space, particularly in terms of creative activity. In our view, silver content meets two criteria of “digital wisdom”: firstly, it results from the life experience of the older people and, secondly, it is published in adequate digital forms of communication where one can expect to reach an audience who can access the content on social media platforms or elsewhere online.

### Action Research as a Source of Insights in Internet Studies

Well-known in educational research, the action research method is located on the borderline between academic work and social activism. It involves the implementation of socially desirable activities while subjecting them to critical consideration in order to improve the quality of its implementation. It involves an effort to examine the studied phenomenon by provoking its existence, empowering participants to undertake joint action, taking place in non-laboratory conditions, for example, in social reality. The concept of the creative process emerging from this experience of collaboration has provided the participants with the label digital sages 80+, which refers to digital wisdom and to the age of people whose creative activity we have observed, inspired, and enhanced.

While the testing in action method adopted here is not perfect, it is difficult to get a better view of the issues of the silver digital economy and the gray digital divide. A description of the path from the gray digital divide to the silver digital economy, including the issue of creative and productive participation of older people in the online space, could be based on existing data. However, if we search for cases involving the effective creation of such content, this would not necessarily provide us with insight into the process of their creation and publishing. Put simply; it is probably more economical and informational to research in action than to look for effectively completed activities with the purpose of describing them.

The purpose of the research in the action method, used primarily in educational research, is in line with the systematic assistance the proxy users provided to two senior citizens, in their individual processes of creative writing and attracting interested audiences through creativity. The people proxy users work with are aged over 85 and have rich, creative professional life stories. Paradoxically, but also in line with the above-mentioned categories of gray digital divide or new-media exclusion (Siapera, 2011), the development of information and communication technologies excluded their free participation in contemporary creative communication. Both authors were able to create the content through their own efforts while additional activities related to publishing were beyond their capabilities.

Publication of a text requires several auxiliary technical activities, for example, selecting a catchy title that will effectively compete for viewers' attention. This calls for familiarity with the nature of online social media. The approach that proxy users have proposed in this particular study in action combines the media- and content-related perspective. Equally interesting in this approach is the extra-textual question, that is the selection of appropriate digital media, as well as the intra-textual issue concerning just the attributes of the content published in a given medium.

### Participants of the Action Research

Two well-educated older people joined the action research: one in 2007 and another in 2012. The first subject, a woman born in 1931, was 75 at the time the research began. The second subject, a man born in 1929, was 83 when joining the action research. This means that the participants grew up in a pre-1939 (e.g., pre-war) Poland, but their potentially critical life events took place during the war and in the post-war People's Republic. They both had successful academic track records in this Central European society, with some exposure to the Western experience. They are considered authorities in their fields but had little experience with new media and Internet-related technology. They were co-initiators of the action. While accessing the action research, they were informed about the academic context of their inclusion into the action. Moreover, they have both willfully become meta-reflective, for example, oriented toward discussing and sharing with both researchers and their readers their emerging creative experience in new media. At the start of the action, this experience was focused on their blogs, but later they changed the digital medium from weblogging to Facebook podcasting or Facebook writing.

The results hereby presented and discussed refer to the 2006 to 2019 period in the case of the first subject and to the 2012 to 2019 period in the case of the second subject. However, 2016 is a landmark because of the change in the Polish digital ecosystem, which resulted in the business-motivated closing of the blogging platform. Both subjects decided to transfer their activities to Facebook. Thus, only the quantitative effects of the research intervention up to 2016 are summarized below.

## Results

The effectiveness of action research is illustrated with three indicators: the qualitative insights, the quantitative description and the social significance of the topics covered in the created content.

### Qualitative Effects of the Intervention

The study in action, in this case, consists of being an editor and quasi-publisher of texts published by authors in a multidimensional space of social media: blogs as well as Facebook or Twitter. Proxy users have created an online environment for older people interested in writing and sharing their work. It allows senior citizens to talk about their lives and share their experience and opinions with members of younger generations. Proxy users heard from the authors that this digital work has an essential function in their lives, including a therapeutic and a compensating function. They receive motivating feedback from readers, often further inspiration, which serves as a challenge, stimulating them to maintain intellectual readiness. However, the benefits are mutual. Such work allows the authors to integrate and reinterpret their life experience but also helps readers develop (as evidenced by numerous reactions and comments with thanks: a total of over 8.8 thousand on one of the blogs). In the comments, the word wisdom is also present, which clearly indicates that this work is perceived in this light. Undoubtedly, this activity also encourages the editor to act as a spokesperson for both sides, linking the author and the reader.

### Quantitative Effects of the Intervention

Both blogs were popular and gained hundreds of thousands of visits (for detailed information see paragraph below). The number of reads and comments for the published content clearly indicates that there is a need for consumption of silver digital content and that digital wisdom can be effectively transmitted using the Internet.

One of the blogs illustrating the concept of silver digital content has existed since June 2007, on a dedicated blog platform. Until October 9, 2016, it recorded over 170,000 reader visits to sub-pages covering a total of 915 entries. On average, one entry has over 180 visits. A total of over one hundred entries are created per year, and since 2016, the publication of texts has been transferred mainly to Facebook, where some entries are displayed more than 10,000 times and generate vivid reactions (likes, sharing, and other signals that the text has been noticed). This disproportion between, on average, merely hundreds of visits to a single blog entry and thousands of visits to Facebook entries illustrates the media change that took place in nearly a decade of the public existence of the blog. The popularity of Facebook as a space for creative writing has outpaced the popularity of dedicated blog platforms.

The other blog does not exist on a separate blogging platform but, instead, within a journalism portal. It has existed since November 2012, and the visitor counting system has registered over 1.48 million visits to a total of 434 entries until 2016. On average, therefore, over 3.4 thousand people read each entry, and more than one hundred entries are created each year. In addition to the blog as a source of visits, posts to a Twitter account are also occasionally sent. However, most traffic to the blog comes from the new entries appearing on the main page of the journalism portal. In 2017 the blogging platform was closed, the content archived, and the publishing transferred to Facebook. It gained popularity there, resulting in two exceptional blog posts read more than 400,000 and 500,000 times.

### Forms and Themes of the Created Content in Relation to Digital Wisdom

One element of the adopted action research approach that was particularly important for the effects of the intervention and for the genres, was the lack of delays in publishing. The texts were released at most within 24 h after their delivery, and usually immediately. It resulted in specific results of the intervention.

One of the first interventions made in both cases took place during the initial phase of each blog: proxy users encouraged both bloggers to devote one entry to one issue. The authors' habits developed when they used to manage regular sections in various periodicals and, as a result, they tended to use the space available at a given moment, without perceiving the digital space as unlimited and divisible. Meanwhile, raising a few topics in one entry resulted in readers being less engaged in the content, for example, there were fewer comments, diverging discussions into several directions, undermining the overall dynamics. In turn, when topics were allocated to separate entries, this boosted readers' response.

Interestingly, neither of the authors wanted to join the discussions under their texts on a regular basis. They only did it occasionally. However, they were happy to comment on the content of the readers' comments in their next entry, summarizing the discussion. Most often, they referred only to selected topics and had no ambition to comment on all comments made by readers. This inspired new discussions, and the process was repeated.

A genre common to both blogs was a journalistic article bordering on a commentary and a column, mostly personal but not jocular. These texts were written in a journalistic style, sometimes in a harsh tone of opposition to reality. These articles were often related to public affairs because both authors devoted their professional lives to working in both formal and informal education (universities, scouts groups, and group animation) and in the health care system (a hospital, a clinic, and a hospice).

During the presented period regular memories also appeared in their works, mostly referring to the current situation and pointing to some analogies to the personal experience of the past. These memories in both blogs reached back to the authors' childhood, for example, to the pre-war years. The authors invoked the Second World War and post-war years, international cooperation (whether academic or ecumenical) in the 1970s and 1980s, as well as the situation of becoming and being an older human. It can be assumed that writing was a function of organizing these experiences.

In both blogs, debates on morality, ethics, axiology, civilization, religion, European values, the problem of anti-Semitism, violence, and collective memory also appeared in the discussed period.

One of the blogs repeatedly revoked the topic of quasi-retreat reflections, which is a record of reading religious texts. This corresponded with the author's professional specialization. The popularization of developmental biomedicine can be considered as its equivalent in the other blog because the other author's professional specialization is related to that area.

The authors' own hobbies were presented by inviting the audience to share the experience. In one case, the author offered recommendations for opera music whereas the other recommended specific reportages.

A rare yet regular type of entry involved farewells, commemorating deceased friends or public figures who were known to the authors.

Whenever the public debate in Poland focused on specific issues related to world-views, both authors were vividly involved in these discussions, usually with a slight delay vs. the press releases. These were critical-political and ideological texts at the time of publication: their usefulness may be evidenced by the fact that readers were interested in discussing their content or sharing them on social media.

The selection of topics by both subjects participating in the research leads to the conclusion that their potential of wisdom has been successfully transferred to the sphere of digital genre-communication forms. In this process, the concept of digital wisdom, as it has described in the introductory sections, has been clearly manifested.

## Further Research Questions and Directions for Further Reflection

Given the above theoretical categories and action research, we formulated four new research questions. They should be considered within interdisciplinary studies at the crossroads of public health, sociological theory, gerontology, and human-computer interaction studies.

### Human Proxy Users Should be Researched in Action in Order to Optimize Non-human Technology-Based Assistants

Given the technological development toward artificial intelligence simulating human behavior and actions, the successful and fruitful cooperation of older creative Web users and human proxy users will become the pattern for advanced technology focused on digital wisdom enhancement.

Both people the proxy user worked with were over 85 years old and had rich creative professional life stories. Stimulation and help in publishing their creative writing such as journalism, non-fiction, and memories allow authors to integrate and reinterpret their life experience. The word wisdom in the term digital wisdom should, therefore, be taken seriously and literally—as real-life wisdom, resulting from experience and reflection, skillfully transferred to the digital world. After the action research was conducted, we identified several interesting areas of further research which are presented below.

By developing the concept of silver content, we would like to suggest analogical research in action for people interested in understanding creative writing. It would be interesting to see how the silver digital content is consumed and integrated into a modern digital workflow media—especially to the social media space.

The next research question pertains to the optimization of the roles of proxy assistants. Since even homo sapiens digital from Prensky's concept (2009)—relatively young, from the academic and public affairs circles—only after 6 years of consultant-mediated presence on Twitter set up his own profile. Thus the digital sage creating silver content—before he or she becomes self-sufficient in its publishing (if at all)—can and should use the support of digitally assisting consultants. It is worth studying the possible need for offering and constantly researching this type of service as a public service.

### Reflection on the Conditions Under Which Technology Can Be a Viable Substitute for Proxy Users

The solutions that enable the creation of silver content can also come from the area of the technology itself. The development of digital technologies prompts us to think about ways in which they can participate in preventing the disengagement and exclusion of older people. So finally, another research question in this area is whether the proxy user that we employed in our action research can be substituted by currently emerging technological solutions that will empower users to create silver digital content autonomously.

In the last decades, digital interfaces have become increasingly simpler. The peak of the process of complexity was programming in assembler, which was then replaced with the DOS operating system, and next—by a graphics system (Windows, iOS). These systems have been modified and further simplified by entering the market of mobile technologies in which, instead of a cursor or a stylus, the finger was used to control. Voice interfaces are the next stage of this process.

The use of simplified interfaces, with particular reference to voice interfaces, makes the technology more accessible to older people. Content and application manufacturers are also beginning to see the growing market of older people, which results in the design of technologies and software aimed at this target group, and takes into account its specificity (weaker sight, weaker motor control). It can, therefore, be said that the threshold of barriers caused by the technology is getting lower. As an example, we can point that as of the end of 2018, a new type of device which incorporates voice assistant technology is announced by major Internet and hardware companies like Facebook and Lenovo. This type of device extends the simple voice assistant that until now was only available in screenless devices (in the form of diverse types of speakers) or as a mobile phone software. The major upgrade vs. current voice assistant hardware is the addition of the screen that allows video-conferencing or posting to social media using voice recognition and speech to text technology. One can easily imagine that such devices could be successfully used by older adults as a tool to generate silver content replacing the current role of the human proxy. This replacement probably couldn't be comprehensive, because voice assistant devices still require installation and configuration. Thus, the role of a proxy would be changed and diminished, but not eliminated in the near future. In the long run however, machines will be able to replace proxy users.

### Voice Assistant Technology Could Become the Main Proxy for Production of Silver Content

The potential use of voice assistant technology as a proxy for production of silver content generates a number of new challenges. Voice assistants have the ability not only to listen to voice commands. Artificial intelligence algorithms on which voice assistants are based today can for example, paraphrase an article from Wikipedia. One can imagine that more advanced algorithms will be able to actively engage in the editing of content. New research questions therefore arise. First, to what extent can an assistant interfere with style? Spoken language is different than written language and interviews printed in the press are not transcribed from a tape but edited and processed from journalistic text. Should voice assistants be something of an advanced voice recorder or rather an intelligent, conscious journalist? Second, to what extent can the assistant correct content (e.g., factual mistakes that can be checked in the encyclopedia, but also about facts from the life of an older person)? Third, to what extent can the assistant give a tone to the content (e.g., emotionally moving, cheerful, playful)?

One can imagine the spectrum of such an assistant's activity from a passive recorder, to a proactive journalist who takes the natural speech of older people as material and constructs something based on this. The question arises as to whether the development of technology will reverse the situation. Finally, intelligent technology may use the ocean of data and experiences produced and recorded by people, and after transforming it into knowledge provide it to recipients.

### Interactive and Intelligent Technology Will be the Substitute for Social Actors That Prevent Exclusion and Disengagement

The above-discussed case is a situation in which technology is a mediator (proxy) between older people and other members of society. Thanks to this, the risk of excluding older people from society is decreasing. One can imagine another role of technology—not as a link to the community, but as a substitute of community.

The phenomenon of treating technologies as social beings was described in the 1990s by Byron Reeves and Clifford Nass in the book “The media equation.” In a series of experiments with computers, they proved that people tend to perceive technology in social categories. Even a computer displaying information on the screen is perceived and described in categories that define people (e.g., malicious, reliable, nice, and friendly) (Reeves and Nass, 1996). This tendency, natural for human perception, is strengthened in the case of voice assistants by the fact that they use natural language and speaking, interacting verbally with the user. After a dozen or so minutes of use, some users tend to treat this technology in terms of talking to a person (e.g., they thank the voice assistant for answering questions and providing information, or they formulate their request in the polite form).

One can imagine virtual assistants as peculiar companions with whom you can talk, ask to tell a joke, sing a song, or check something in Wikipedia. The advantage of such a virtual companion would be proactivity (in contrast to today's radio or television, which also serve as a fulfillment of silence). In comparison with other people—an advantage may be continuous availability. A virtual companion always has time and can give the user his or her attention; he or she will never excuse himself or herself from the conversation. Now voice assistants can substitute the social interaction in a very restricted manner, but with the development of this technology, these interactions can become more meaningful and may become a way to provide aging people with the opportunity to maintain social interactions on the level necessary to stay active.

## Author Contributions

All authors listed have made a substantial, direct and intellectual contribution to the work, and approved it for publication.

### Conflict of Interest Statement

The authors declare that the research was conducted in the absence of any commercial or financial relationships that could be construed as a potential conflict of interest.
